# Coping and behavior as predictors of childhood anxiety: an SEM-based analysis in school-going children

**DOI:** 10.3389/fpsyt.2026.1759096

**Published:** 2026-02-20

**Authors:** Archana Chhetri, Samrat Singh Bhandari, Sonam Ongmu Lasopa, Sanjiba Dutta

**Affiliations:** 1 Department of Psychiatry, New Sir Thutop Namgyal Memorial Hospital, Gangtok, Sikkim, India; 2 Department of Psychiatry, Sikkim Manipal Institute of Medical Sciences, Sikkim Manipal University, Tadong, Sikkim, India

**Keywords:** anxiety, behavioral regulation, child mental health, coping strategies, emotional and behavioral, prosocial behavior, school children, structural equation modelling

## Abstract

**Introduction:**

Childhood anxiety is a growing concern in Indian school settings, yet the behavioral and coping mechanisms that shape anxiety outcomes remain insufficiently understood. This study examined how coping patterns, behavioral tendencies, prosocial characteristics, and socioeconomic factors predict anxiety among school-going children in Sikkim.

**Methods:**

A cross-sectional survey was conducted among 1,001 children aged 7 to 10 years in Sikkim. Standardized instruments were used, including the Revised Children’s Manifest Anxiety Scale-2 (RCMAS-2), the Strengths and Difficulties Questionnaire (SDQ-P), the Coping Strategies Inventory (CSI-SF), and the 2020 Kuppuswamy Socioeconomic Scale. Analyses included descriptive statistics, Chi-square tests, correlations, regression models, and structural equation modelling.

**Result:**

Based on established RCMAS-2 thresholds, 22.5% of children screened positive for clinically elevated anxiety (21.0% moderate; 1.5% severe). In the structural model, maladaptive coping showed the strongest association with anxiety (β = 0.18, p < 0.001). Adaptive coping (β = −0.08, p = 0.057) and prosocial behavior (β =-0.11, p = 0.035) showed small protective effects. Internalizing and externalizing behavior showed weak direct associations with anxiety and minimal indirect effects through maladaptive coping. Overall, behavioral and coping variables exhibited low intercorrelations, indicating that anxiety symptoms were associated with coping patterns rather than broad behavioral difficulties.

**Conclusion:**

A substantial proportion of primary-school children in Sikkim exhibit clinically elevated anxiety symptoms. Coping style, particularly maladaptive coping, emerged as the most salient factor associated with anxiety, whereas behavioral difficulties contributed minimally. Strengthening adaptive coping and encouraging prosocial engagement within school mental health programs may help reduce anxiety risk during early schooling years.

## 1 Introduction

Anxiety is one of the most common emotional difficulties during childhood and early adolescence, influencing psychological well-being, academic participation, and social functioning (1, 2). Global estimates indicate that between 10-20% of school-aged children experience significant anxiety symptoms, many of which remain unrecognized or untreated (3–5). In India, growing academic pressure, increased social comparison, and changing family dynamics have contributed to rising rates of anxiety among children (6, 7). These concerns are particularly visible in regions where scholastic performance carries strong social value (8). Despite this, research on the behavioral and coping factors linked to childhood anxiety in Indian settings remains limited, especially in smaller Himalayan states such as Sikkim (6, 9, 10). Childhood anxiety is characterized by physiological arousal, worry, and avoidance (11). Developmental models suggest that these symptoms arise through the interaction of temperament, stress exposure, and learned responses (12). Within school settings, behavioral tendencies are often described along two broad dimensions: internalizing and externalizing behaviors (13). Internalizing behaviors include fearfulness, withdrawal, and emotional distress, whereas externalizing behaviors involve outwardly directed responses such as impulsivity or conduct-related difficulties. Although both patterns are relevant for adjustment, internalizing tendencies show a more consistent association with anxiety symptoms across childhood (14–16). Understanding how these behavioral domains relate to anxiety in school environments is important for early identification and support (17, 18).

Coping strategies play a central role in how children manage stress and emotional challenges. Coping has been defined as the cognitive and behavioral efforts used to manage demands perceived as taxing or overwhelming (19). Adaptive strategies, including problem solving, cognitive reframing, and seeking support, are generally associated with better emotional adjustment (20, 21). In contrast, maladaptive coping strategies such as avoidance, rumination, and wishful thinking often maintain or increase anxiety levels (14). While international research has documented these associations, few studies from India have examined coping patterns alongside behavioral tendencies when explaining childhood anxiety (6, 22, 23). Prosocial behavior is another important factor in children’s emotional development. Acts such as sharing, cooperation, and helping others promote stronger peer relationships and higher emotional competence (24, 25). Evidences suggest that children who engage in prosocial behavior tend to show better emotional adjustment and lower stress (26). In collectivist contexts like India, where social connectedness is strongly valued, prosociality may play an even greater role, yet there is limited empirical evidence from smaller states and rural–urban mixed populations (6, 27).

Socioeconomic status also shapes children’s coping resources and emotional resilience. The Kuppuswamy scale, which combines education, occupation, and income, is widely used to classify socioeconomic background in India (28–31). Lower socioeconomic standing is often associated with higher stress exposure and fewer coping resources (32). However, higher socioeconomic standing does not uniformly protect against anxiety, since high expectations in competitive settings can contribute to pressure (6, 7). This complexity highlights the importance of examining socioeconomic influences within specific regional contexts (10). Sikkim offers a distinctive setting for examining childhood anxiety, given its geographic isolation, strong emphasis on academic attainment, and limited availability of child-focused mental health services (33). Despite these features, systematic data on anxiety and its psychosocial correlates among school-going children in the state are scarce.

The present study was designed to address this gap by integrating emotional, behavioral, and coping dimensions into a unified analytical framework. Using multivariate analyses and structural equation modelling, the study sought to: (1) estimate the prevalence and severity of childhood anxiety using the RCMAS-2 (11); (2) examine how internalizing, externalizing, prosocial behavior, and coping patterns relate to anxiety; and (3) test a structural model assessing direct and indirect pathways linking behavioral tendencies, coping style, and contextual factors to anxiety outcomes.

## 2 Materials and methods

### 2.1 Study design and setting

This cross-sectional, observational study was conducted among school-going children in the state of Sikkim, India, with the aim of identifying the interplay between coping styles, behavioral regulation, prosocial traits, and anxiety. The investigation combined quantitative psychometric assessment with multivariate statistical modelling. Data were collected from March 2018 to November 2019 across all four administrative districts: East, West, North, and South to ensure representation of both urbanized and rural schooling environments.

### 2.2 Study population and sampling strategy

**Figure 1:**
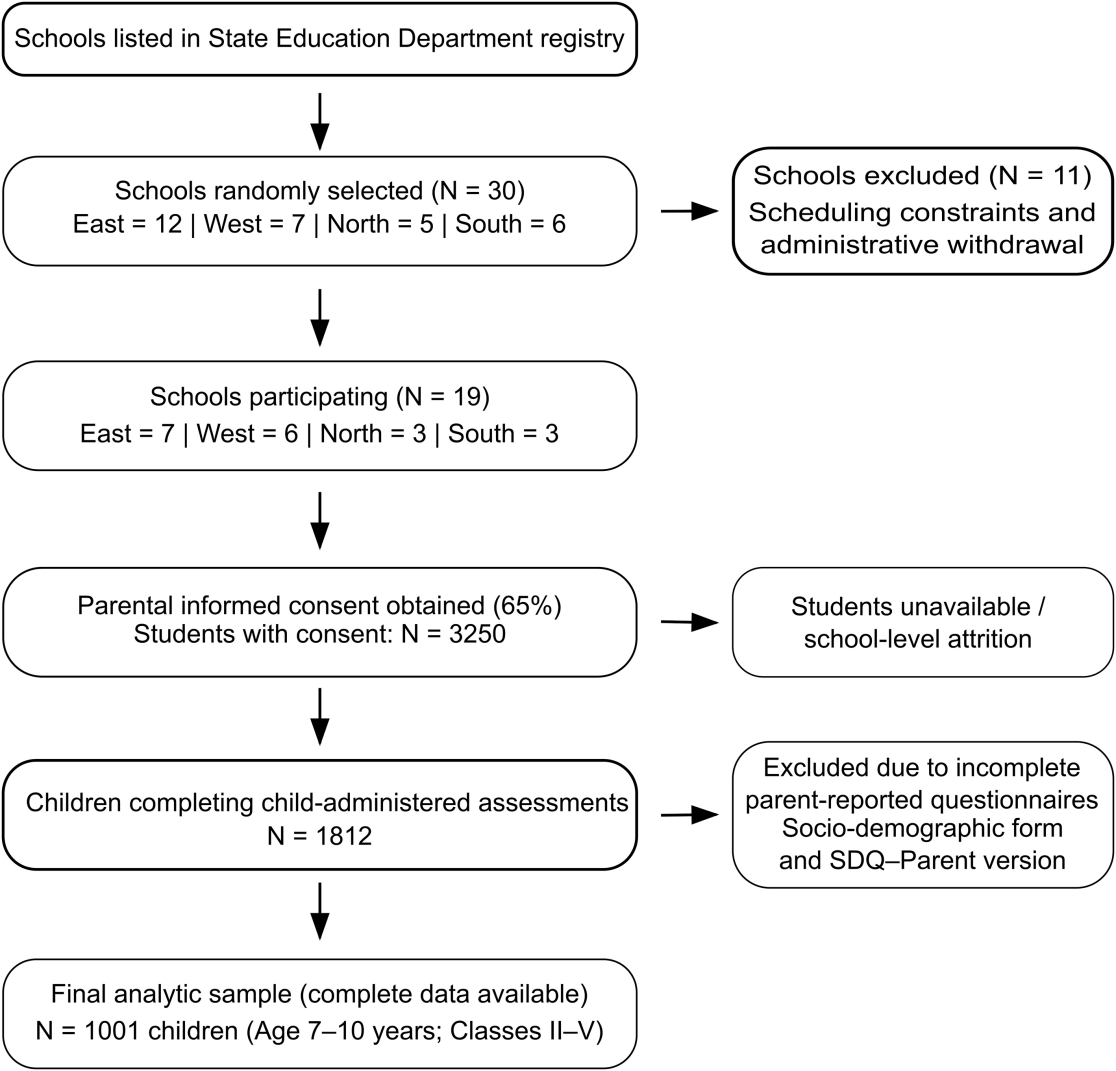
Full Spearman correlation heatmap including RCMAS-2 total raw score. Heatmap showing correlations among all behavioral, coping, and anxiety-related parameters: Class, Age, Gender, RCMAS-2 Total Raw Score, Coping Strategies Inventory subscales including Problem Solving, Cognitive Restructuring, Expressing Emotions, Social Support, Problem Avoidance, Wishful Thinking, Self-Criticism, Social Withdrawal, Primary-level adaptive coping domains (PFE, EFE, PFD, EFD), Total Engagement (Eng.), Total Disengagement (Dis.), SDQ-P domains (EPS, CPS, HS, PPE, Prosocial), and composite SDQ-P indices Externalizing (EXT) and Internalizing (INT).

A list of all private and public schools registered with the State Education Department was obtained, and 30 schools were randomly selected across districts (East = 12, West = 7, North = 5, South = 6). Of these, 11 schools did not participate due to administrative constraints, including scheduling difficulties and withdrawal citing time limitations, resulting in participation from 19 schools (East = 7, West = 6, North = 3, South = 3). Written informed consent was obtained for 65% of eligible students (N = 3250). Of these, approximately 40% were not available for assessment due to school-level attrition or absence on the day of data collection, yielding 1812 children who completed child-administered assessments. Complete data on all study variables were available for 1001 children. The primary source of missing data was non-return of parent-completed questionnaires, specifically the socio-demographic form and the Strengths and Difficulties Questionnaire–Parent version. As these measures were required for behavioral and socioeconomic analyses, cases with incomplete parent-reported data were excluded from the final analytic sample using listwise deletion. Comparisons of available demographic characteristics (age, gender, and district) between included and excluded participants did not indicate marked differences, although differential nonresponse related to socioeconomic factors cannot be ruled out. This potential for systematic bias is acknowledged and considered when interpreting the findings. The final sample consisted of 1001 children aged 7–10 years enrolled in classes II–V in English-medium schools recognized by the Department of School Education, Government of Sikkim (Selection process detailed in **Figure 1**). A multistage stratified random sampling approach was used, with schools selected through proportional allocation based on district-level enrollment. Eligibility criteria included enrolment as a full-time student within the specified age range, ability to understand instructions in English or Nepali, and provision of parental consent and child assent. Children with diagnosed neurodevelopmental disorders, sensory impairments, or chronic medical conditions likely to affect behavioral assessment were excluded.

### 2.3 Assessment tools

The primary anxiety outcome was measured using the Revised Children’s Manifest Anxiety Scale–Second Edition (RCMAS-2; 11). The scale consists of 49 items scored dichotomously (“Yes/No”), producing subscale raw scores for Physiological Anxiety, Worry/Oversensitivity, Social Concerns/Concentration, and two validity indicators (Defensiveness, Inconsistent Responding), along with a Total Anxiety Raw Score. In the present study, analyses were performed using raw scores, and severity classification was based on U.S. normative T-score conversions provided in the official RCMAS-2 manual, as no Indian population norms are currently available. Cut-offs for interpretive categories (e.g., typical, mild, moderate, severe) were therefore derived using the manual’s age-based T-score ranges.

Behavioral characteristics were assessed using the Strengths and Difficulties Questionnaire–Parent Version (SDQ-P; 34, 35), generating scores for internalizing, externalizing, and prosocial behavior. Coping was measured using the Coping Strategies Inventory–Short Form (CSI-SF; 36), which yields adaptive and maladaptive coping indices based on eight domain scores. Socioeconomic status was assessed using the Kuppuswamy Scale (2020), and demographic variables were retained as covariates (28). For analysis, we computed Spearman correlations, hierarchical multiple regression models, mediation with bootstrap confidence intervals, and structural equation modelling (SEM). Prior to SEM, measurement models for latent constructs derived from SDQ-P and CSI-SF were evaluated to ensure acceptable factor structure and reliability.

### 2.4 Statistical analysis

Data were analyzed using Python, RStudio, MS Excel, and GraphPad Prism 7. Descriptive statistics summarized demographic variables, SES distribution, and mean scores of all scales. Categorical associations were examined using Chi-square tests. Spearman correlation coefficients assessed relationships among continuous variables. To identify predictors of anxiety, multiple linear regression models were estimated using the RCMAS-2 total raw score as the dependent variable. Independent variables included internalizing and externalizing behavior, adaptive and maladaptive coping, prosocial behavior, age, gender, class level, and SES. Before modelling, multicollinearity was evaluated using Variance Inflation Factors (VIFs), and all predictors had VIFs below 5, indicating acceptable levels of collinearity. Although the Kuppuswamy Scale is ordinal, its three components (education, occupation, income) generate a composite score with quasi-continuous properties; therefore, SES was treated as a continuous predictor to preserve variability and statistical power, consistent with prior Indian research using multidimensional SES indices in child and adolescent mental health studies. For higher-order interpretation of pathways, Structural Equation Modelling (SEM) was performed (37). For SEM analysis, confirmatory factor analysis (CFA) was conducted to evaluate the measurement structure of the latent constructs included in the model. CFA was performed at the subscale level. Adaptive and maladaptive coping were modelled as separate latent constructs using Coping Strategies Inventory–Short Form (CSI-SF) subscale scores. Adaptive coping was indicated by problem solving, cognitive restructuring, emotional expression, and social support, while maladaptive coping was indicated by problem avoidance, wishful thinking, self-criticism, and social withdrawal. Behavioral tendencies were modelled using Strengths and Difficulties Questionnaire–Parent version (SDQ-P) subscales, with internalizing behavior indicated by emotional and peer problems and externalizing behavior indicated by conduct problems and hyperactivity. Anxiety was modelled as a latent construct using RCMAS-2 domain scores. Latent constructs for Behavior, Coping and Anxiety were developed based on theoretical considerations and correlation patterns. The model was estimated using maximum likelihood with robust standard errors. Model fit was evaluated using standard indices, including the Comparative Fit Index (CFI), Tucker–Lewis Index (TLI), Root Mean Square Error of Approximation (RMSEA) and Standardized Root Mean Square Residual (SRMR). Indirect effects were tested using 2,000-iteration bias-corrected bootstrap procedures, and mediation effects were interpreted using standardized coefficients with 95% confidence intervals.

### 2.5 Ethical considerations

The study involved human participants and was reviewed and approved by the Institutional Ethics Committee and the Institutional Review Committee of Sikkim Manipal Institute of Medical Sciences, Sikkim Manipal University. Written permission was obtained from the participating schools prior to data collection. Written informed consent was obtained from the parents or legal guardians of all participating children, and verbal assent was obtained from the children themselves prior to assessment. All study procedures were conducted in accordance with applicable institutional guidelines and ethical standards for research involving human participants.

## 3 Results

### 3.1 Participant characteristics

The study included 1,001 school-going children aged 7–10 years (M = 8.6, SD = 1.1) from Sikkim’s four districts, with a near-even gender distribution (52.8% male, 47.2% female). Socioeconomic status (SES) per the 2020 Kuppuswamy Scale showed 17.5% upper class, 33.4% upper-middle, 26.7% lower-middle, 18.2% upper-lower, and 4.9% lower class, with significant district-wise variation (χ² = 23.64, df = 12, p = 0.022). East Sikkim had the highest upper-class representation (19.5%), while North Sikkim had the most lower-middle families (38.5%). Parental education and occupation correlated positively with income (r = 0.48–0.61, p < 0.01). (**Tables 1**, **2**).

**Table 1:** Demographic characteristics of the study population.

Characteristic	Category	n	%
Grade	Grade 2	302	30.2
	Grade 3	356	35.6
	Grade 4	245	24.5
	Grade 5	198	19.8
Gender	Male	480	48.0
	Female	521	52.0
Religion	Buddhist	240	24.0
	Christian	110	11.0
	Hindu	610	61.0
	Islam	30	3.0
	Others	11	1.1
RCMAS-2 Anxiety Severity	Negligible (<39)	83	8.29
	Mild (40–60)	693	69.23
	Moderate (61–70)	210	20.98
	Severe (≥71)	15	1.50
Socioeconomic Status (Kuppuswamy 2020)	Upper	174	17.4
	Upper-Middle	334	33.4
	Lower-Middle	267	26.7
	Upper-Lower	182	18.2
	Lower	44	4.4
District	East	327	32.7
	West	236	23.6
	North	224	22.4
	South	214	21.4

**Table 2:** Distribution of respondents across socioeconomic status (Kuppuswamy Scale 2020).

District	Upper (17.5%)	Upper-Middle (33.4%)	Lower-Middle (26.7%)	Upper-Lower (18.2%)	Lower (4.9%)	Total (N)
East (327)	57	109	87	59	15	327
West (236)	41	79	63	43	10	236
North (224)	39	75	60	41	09	224
South (214)	37	71	57	39	10	214
All Districts	174 (17.4%)	334 (33.4%)	267 (26.7%)	182 (18.2%)	44 (4.4%)	1001

### 3.2 Prevalence and patterns of anxiety

69.2% of children were classified as having mild anxiety, 21.0% moderate, and 1.5% severe as RCMAS-2 scale. Anxiety scores were significantly higher in East and South districts compared to North and West (p < 0.05). Anxiety increased with age (r = 0.10, p < 0.05), but gender differences were non-significant.

### 3.3 Behavioral and prosocial characteristics

The Strengths and Difficulties Questionnaire (SDQ-P) indicated higher internalizing (emotional and peer problems) than externalizing (conduct and hyperactivity) scores, with 39.6% of children in the borderline/abnormal range for total difficulties. Prosocial behavior was inversely associated with total difficulties (r = -0.41, p < 0.001) and anxiety (r = -0.04, p < 0.05). District differences in SDQ-P scores were significant (p < 0.05), but gender and SES showed minimal impact. Internal consistency was acceptable (Cronbach’s α = 0.82) (**Table 3**).

**Table 3:** Family demographics vs behavioral & coping outcomes.

Variable	EXT	INT	Total_Eng	Total_Dis
No of Siblings	0.03	0.02	0.05	0.01
Father education	−0.06	−0.15	0.01	0.03
Mother’s Education	0.04	0.10	0.03	0.01
Family Annual Income in lakhs	−0.06	−0.16	−0.01	−0.01
Age	−0.06	NS	NS	NS
Gender	−0.08	NS	NS	NS

### 3.4 Coping strategies and associations with anxiety

**Figure 2:**
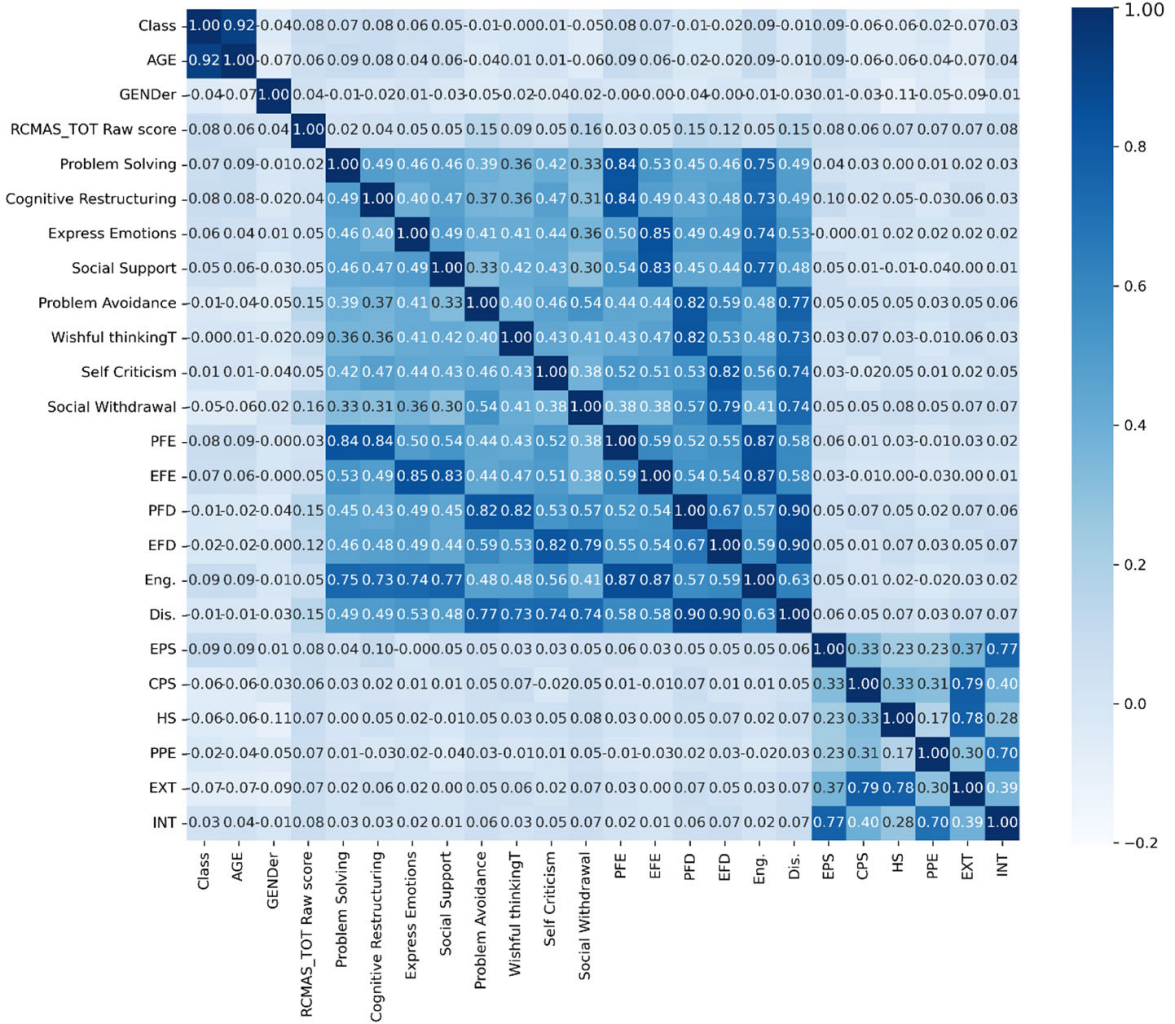
Summary correlation heatmap for crucial variables. Heatmap showing inter-correlations among the primary predictors included in the structural equation model: Internalizing Behavior (INT), Externalizing Behavior (EXT), Total Adaptive Coping (Eng.), Total Maladaptive Coping (Dis.), and RCMAS-2 Total Score.

**Figure 3:**
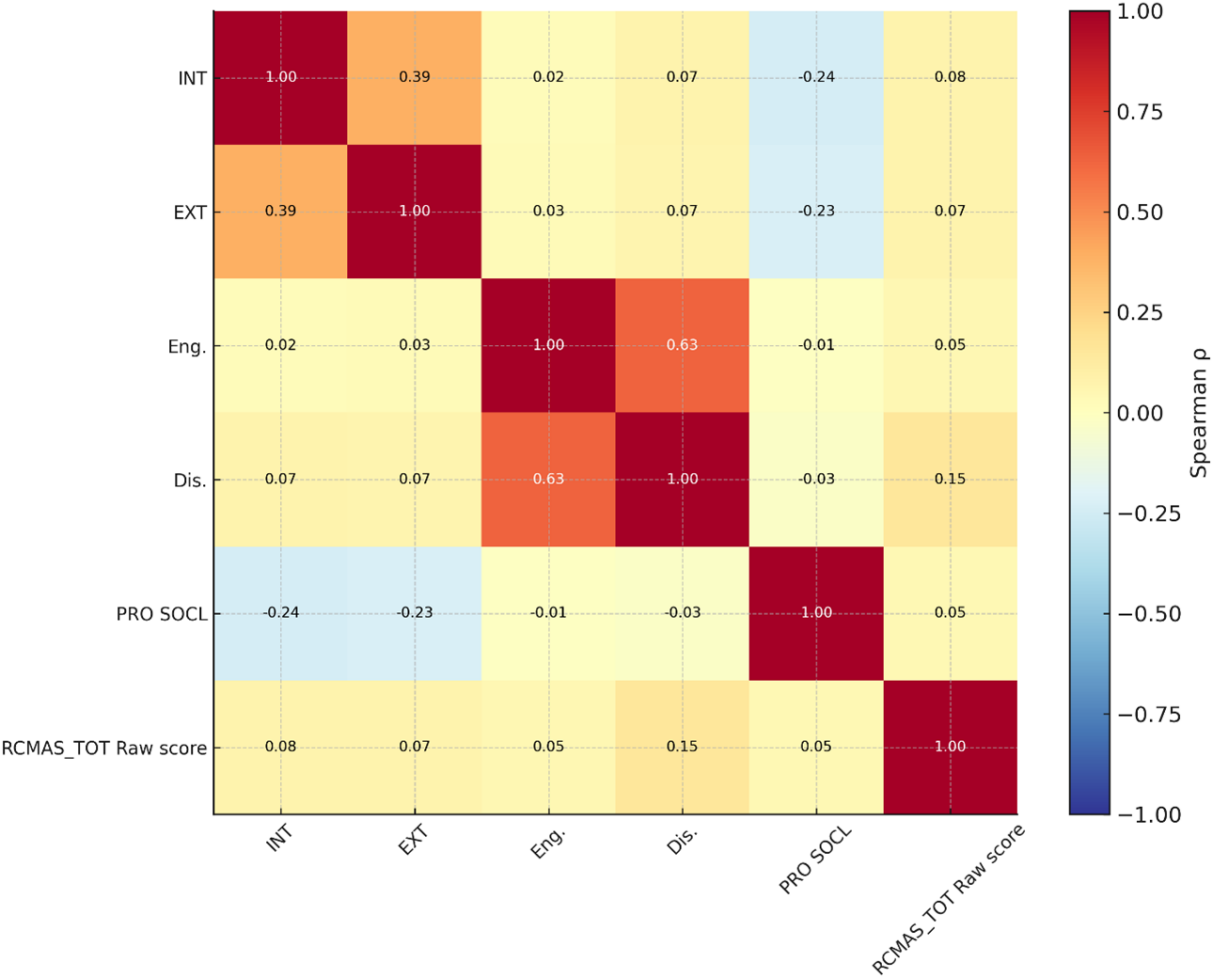
Structural equation model predicting anxiety. Structural Equation Model (SEM) depicting standardized path coefficients (β), p-values, and bootstrap confidence intervals for associations among Prosocial Behavior (PRO), Internalizing Behavior (INT), Externalizing Behavior (EXT), Total Adaptive Coping (Eng.), Total Maladaptive Coping (Dis.), and RCMAS-2 Total Raw Score (RCMAS). Indirect effects for INT to Dis to RCMAS and EXT to Dis to RCMAS are presented with 95% bootstrap confidence intervals.

The Coping Strategies Inventory (CSI-SF) showed strong internal consistency (α = 0.45–0.88). Maladaptive coping strategies (problem avoidance, wishful thinking, self-criticism, social withdrawal) were positively correlated with each other (r = 0.52–0.87) and with internalization (r = 0.48, p < 0.001). Adaptive coping strategies (problem-solving, cognitive restructuring, emotional expression, social support) were also intercorrelated (r = 0.46–0.75) but showed weaker associations with internalization (r = 0.33, p < 0.01). Maladaptive coping was the strongest correlate of RCMAS-2 scores (r = 0.15, p < 0.01), followed by internalization (r = 0.08, p < 0.05) and externalization (r = 0.07, p < 0.05) (**Tables 4**–**6**). Adaptive coping and prosocial behavior had negligible or inverse correlations with anxiety (r = -0.05 and -0.04, respectively). (**Figures 2**, **3**).

**Table 4:** Chi-square association of SDQ-P with demographic variables.

Variable	χ²	P-value	Df
District	13.234	0.0395*	6
Class	5.085	0.7485	8
Gender	6.451	0.0397*	2
Age Group	0.202	0.904	2

**Table 5:** Chi-square analysis showing the correlation between internalization and externalization behavior across demographic parameters.

Variable	Type	Chi^2^	P-value	Df
District	Externalization	8.76	0.1875	6
District	Internalization	12.386	0.0539	6
Class	Externalization	8.49	0.3871	8
Class	Internalization	6.354	0.6077	8
Gender	Externalization	14.782	0.0006**	2
Gender	Internalization	4.139	0.1262	2
Age Group	Externalization	2.823	0.2437	2
Age Group	Internalization	3.018	0.2211	2

**Table 6:** Linear regression between behavioral outcomes and family demographic variables as predictors.

Outcome	R²	Adj. R²	F-statistic	p-value (F)
Externalization (EXT)	0.013	0.005	1.57	0.1296
Internalization (INT)	0.045	0.037	5.66	0.000**
Prosocial Behavior	0.015	0.007	1.89	0.0582
Total Engagement	0.009	0.000	1.05	0.3937
Total Disengagement	0.006	-0.003	0.67	0.7159

### 3.5 Structural equation modeling

**Figure 4:**
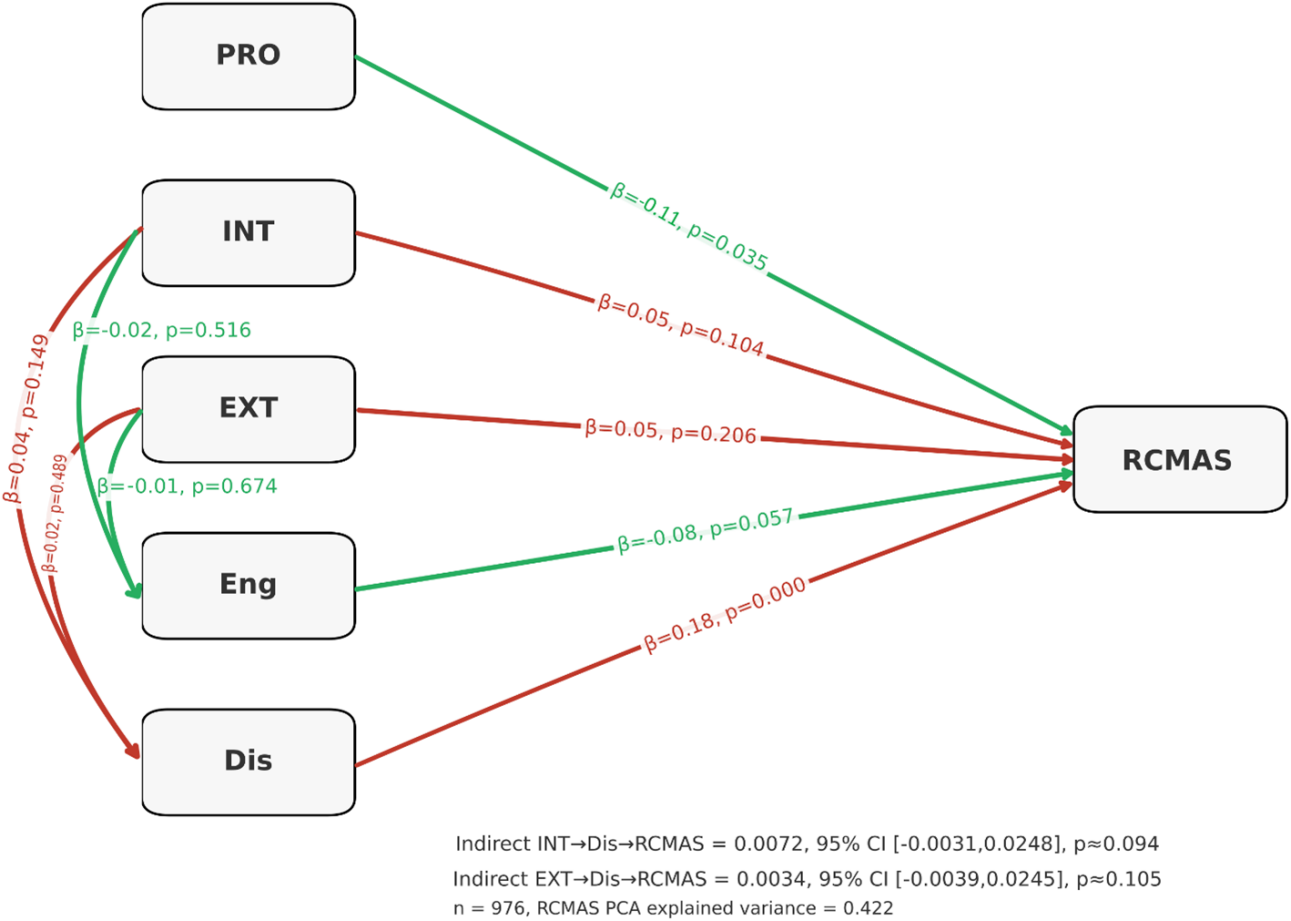
Structural equation model predicting anxiety. Structural Equation Model (SEM) illustrating standardized path coefficients (β values), p-values, and bootstrap confidence intervals for the relationships among Prosocial Behavior (PRO), Internalizing Behavior (INT), Externalizing Behavior (EXT), Adaptive Coping (Engagement; Eng.), Maladaptive Coping (Disengagement; Dis.), and RCMAS-2 Total Raw Score (RCMAS). Indirect effects for INT → Dis → RCMAS and EXT → Dis → RCMAS are shown with 95% bootstrap confidence intervals.

The structural equation model assessing predictors of RCMAS-2 total anxiety score showed that maladaptive coping was the only significantly associated with anxiety (β = 0.18, p < 0.001). Adaptive coping demonstrated a small inverse trend (β = –0.08, p = 0.057), followed by prosocial behavior (β = -0.11, p = 0.035). Internalization (β = 0.05, p = 0.104) and externalization (β = 0.05, p = 0.206) did not exhibit significant direct effects on anxiety. Internalization predicted higher maladaptive coping (β = 0.04, p = 0.149), and externalization showed no meaningful influence on either coping domain (Engagement: β = 0.01, p = 0.674; Disengagement: β = 0.02, p = 0.489). As a result, the indirect pathways linking internalization and externalization to anxiety through maladaptive coping were small and not statistically significant (INT → Dis → RCMAS: indirect β = 0.0072, p ≈ 0.094; EXT → Dis → RCMAS: indirect β = 0.0034, p ≈ 0.105). Overall, the model indicates that maladaptive coping is the primary mechanism associated with elevated RCMAS-2 anxiety, while internalizing and externalizing behaviors contribute weak and largely indirect influences. The PCA-derived RCMAS factor explained 42.2% of variance (**Figure 4**, **Supplementary Table 1**).

Confirmatory factor analysis demonstrated acceptable measurement properties for all latent constructs included in the structural model. Standardized factor loadings for adaptive coping ranged from 0.61 to 0.67, and for maladaptive coping from 0.54 to 0.73, indicating moderate to strong representation of the respective constructs by their indicators. Anxiety domain scores showed strong loadings on the latent anxiety factor, ranging from 0.69 to 0.81. Internalizing and externalizing behavioral constructs demonstrated modest statistically significant, with standardized estimates ranging from 0.43 to 0.49 for internalizing behavior and 0.43 to 0.76 for externalizing behavior. These findings indicate that behavioral tendencies were measured with adequate but lower precision relative to coping and anxiety constructs. Overall model fit indices indicated acceptable fit of the measurement model, supporting the use of the specified latent constructs in subsequent structural equation modelling (**Supplementary Table 2**, **3**). The relative strength of factor loadings across constructs was consistent with the magnitude of path coefficients observed in the structural model.

## 4 Discussion

This study examined how behavioral tendencies, coping style, prosocial traits, and socioeconomic background jointly relate to anxiety as measured by the RCMAS-2 in a large sample of primary-school children in Sikkim. Replacing previously used adult-oriented measures with the RCMAS-2 anchors the results more directly in child-normed anxiety assessment and clarifies how cognitive, somatic, and behavioral components of anxiety interface with coping and conduct variables in this population (11). Overall, maladaptive coping emerged as the dominant proximal correlate of anxiety, with evidence that internalizing symptoms operate partly through maladaptive strategies to increase anxiety levels (14, 20). Adaptive coping and prosociality had consistent inverse associations with RCMAS-2 scores, indicating potential protective mechanisms (21, 24).

The prevalence and distributional findings show substantial anxiety symptomatology across districts and grades, with higher scores concentrated in more urbanized districts. These district differences likely reflect a combination of environmental stressors, such as academic pressure and changing family dynamics, and the differential availability of supportive resources (7, 32). Socioeconomic indicators did not show a direct effect on anxiety but were associated with coping patterns and internalizing behavior, suggesting an indirect pathway. Families with greater educational and occupational resources tended to report coping styles that buffered emotional difficulties, whereas lower socioeconomic status aligned with higher maladaptive coping and emotional dysregulation (38, 39). This pattern highlights the importance of viewing socioeconomic status as a contextual factor shaping the development and deployment of coping, rather than a uniform predictor of anxiety severity (40). The prevalence estimates reported in this study should be interpreted with caution, as severity classification was based on U.S.-derived RCMAS-2 T-score cut-offs in the absence of Indian normative data. Cultural and contextual differences in emotional expression, parental reporting styles, and school-related stressors may influence score distributions, raising the possibility that U.S. norms could overestimate symptom severity in Indian school populations. Consequently, the proportion of children classified in the mild anxiety range is best understood as a screen-positive estimate rather than a diagnostic prevalence. Potential misclassification at lower severity thresholds cannot be excluded, particularly in settings where normative expectations for academic performance and behavioral regulation differ from those in the reference population. The findings underscore the need for future work to develop population-specific normative data for child anxiety measures in Indian settings to improve interpretive precision.

The structural equation model evaluated hypothesized directional pathways among key variables. Maladaptive coping had the largest standardized path coefficient to RCMAS-2, identifying it as a primary proximal risk factor (15). Internalizing and externalizing behaviors exerted a smaller positive association with anxiety; importantly, internalizing behavior was positively associated with maladaptive coping and thereby produced a measurable indirect effect on overall anxiety (41, 42). Adaptive coping and prosocial behavior reduced RCMAS scores, with adaptive strategies showing a stronger inverse effect than prosociality. These pathways point to a mediational framework in which behavioral tendencies influence choice of coping, which in turn modulates anxiety expression (20, 43). The correlation analyses complement the structural equation model by identifying which specific coping components align most strongly with anxiety. Problem-solving, cognitive restructuring, and social support domains correlated negatively with the RCMAS-2 total score, whereas avoidance, wishful thinking, and self-critical strategies correlated positively. This pattern reinforces the conceptual distinction between engagement-oriented (approach) coping that facilitates emotional regulation, and disengagement or rumination-based strategies that maintain physiological arousal and worry (15, 21). Although some associations, particularly between behavioral tendencies and anxiety, were small in magnitude, such effect sizes are expected in multifactorial psychosocial outcomes influenced by overlapping individual, familial, and contextual factors. In this context, the relative strength and consistency of associations across models are more informative than absolute effect size alone.

Prosocial behavior emerged as a modest but consistent protective factor. Children with higher prosocial scores showed lower RCMAS-2 scores and weaker internalization-anxiety links, suggesting that empathic engagement and peer cooperation buffer the psychological impact of stress (44–47). This protective role suggests utility for school-based programs that foster cooperative activities and emotional literacy. The findings carry clear practical implications. Interventions that reduce maladaptive coping and strengthen adaptive strategies should be prioritized. Cognitive-behavioral approaches that teach problem-solving, cognitive restructuring, and social support seeking are likely to be beneficial, especially when embedded in school settings (48, 49). Prosocial skills training and peer-mediated support programs may offer complementary protective effects by increasing the social resources available to children (26, 47). Socioeconomic and district differences highlight the need for context-sensitive programming, particularly in urbanized districts where academic stress is higher. The findings highlight the potential value of school-based interventions that target maladaptive coping strategies while strengthening adaptive coping and prosocial behaviors. Low-intensity, curriculum-integrated programs focusing on emotional regulation, problem-solving skills, and peer support may be particularly feasible in school settings and could contribute to early identification and mitigation of anxiety-related difficulties among children.

## 5 Conclusions, limitations, and future directions

The findings of this study indicate that anxiety among primary-school children in Sikkim is influenced most strongly by coping patterns, with maladaptive coping showing a significant association with higher RCMAS-2 scores and adaptive coping and prosocial behavior offering modest protective effects. Internalizing and externalizing tendencies contributed only weakly, suggesting that coping style, rather than general behavioral difficulty, is the primary mechanism underlying anxiety vulnerability in this age group. These results should be interpreted with caution due to several limitations. The cross-sectional design restricts causal inference, and reliance on self-report may introduce bias, particularly in the absence of parent and teacher perspectives. The sample represents a single Himalayan state and may not fully reflect patterns present in other cultural or educational contexts. The study sample was drawn exclusively from English-medium schools, which may limit generalizability to children attending government-run or non-English-medium institutions where educational environments and stressors may differ. Additionally, findings from Sikkim should be extrapolated cautiously to other regions of India with differing sociocultural and educational contexts. Future work should incorporate longitudinal designs, to examine developmental trajectories and temporal relationships among coping strategies, behavioral tendencies, and anxiety. Incorporating multi-informant approaches, including teacher reports and observational measures, as well as intervention studies targeting coping skills and prosocial behavior, would further strengthen the evidence base and inform preventive strategies in school contexts.

## Data Availability

The original contributions presented in the study are included in the article/**Supplementary Material**. Further inquiries can be directed to the corresponding author/s.
