# Supplementary material for: Coping and behavior as predictors of childhood anxiety: an SEM-based analysis in school-going children
Source: Front Psychiatry. 2026 Feb 20;17:1759096. doi: 10.3389/fpsyt.2026.1759096 (PMC12963723; doi:10.3389/fpsyt.2026.1759096)
Supplement: Supplementary file 1 [file Table1.docx]

| **Total Effects in the SEM Predicting RCMAS-2 Anxiety Scores** | | | |
| --- | --- | --- | --- |
| **Path** | **Standardised β** | **95% Bootstrap CI** | **p-value** |
| **PRO → RCMAS** | −0.11 | [−0.21, −0.01] | 0.035 |
| **INT → RCMAS** | 0.05 | [−0.01, 0.12] | 0.104 |
| **EXT → RCMAS** | 0.05 | [−0.03, 0.14] | 0.206 |
| **Eng → RCMAS** | −0.08 | [−0.17, 0.002] | 0.057 |
| **Dis → RCMAS** | 0.18 | [0.12, 0.25] | <0.001 |
| **INT → Eng** | −0.02 | [−0.09, 0.05] | 0.516 |
| **EXT → Eng** | 0.01 | [−0.06, 0.07] | 0.674 |
| **INT → Dis** | 0.04 | [−0.02, 0.11] | 0.149 |
| **EXT → Dis** | 0.02 | [−0.04, 0.12] | 0.489 |
| **Indirect (Mediated) Effects** | | | |
| **Mediation Path** | **Indirect Effect (a×b)** | **95% Bootstrap CI** | **p-value** |
| **INT → Dis → RCMAS** | 0.0072 | [−0.0031, 0.0248] | 0.094 |
| **EXT → Dis → RCMAS** | 0.0034 | [−0.0039, 0.0245] | 0.105 |
| **Model Fit Indices** | | | |
| **Index** | **Value** | **Interpretation** | |
| CFI | 0.96 | Good fit | |
| TLI | 0.94 | Good fit | |
| RMSEA | 0.041 | Good fit | |
| χ²/df | ~1.9 | Adequate fit | |

**Supplementary Table 1:** Standardized Direct, Indirect, and Total Effects in the SEM Predicting RCMAS-2 Anxiety Scores

**Supplementary Table 2: Confirmatory Factor Analysis (CFA) Standardized Loadings for Latent Constructs Used in SEM**

| **Latent Construct** | **Indicator (Subscale)** | **Standardized Loading (λ)** | **SE** | **z** | **p-value** | **95% CI** |
| --- | --- | --- | --- | --- | --- | --- |
| **Adaptive Coping** | Problem Solving | 0.674 | 0.036 | 18.48 | <0.001 | 0.602 – 0.745 |
|  | Cognitive Restructuring | 0.647 | 0.033 | 19.81 | <0.001 | 0.583 – 0.711 |
|  | Emotional Expression | 0.659 | 0.040 | 16.26 | <0.001 | 0.579 – 0.738 |
|  | Social Support | 0.611 | 0.039 | 15.82 | <0.001 | 0.535 – 0.686 |
| **Maladaptive Coping** | Problem Avoidance | 0.732 | 0.027 | 26.67 | <0.001 | 0.678 – 0.786 |
|  | Wishful Thinking | 0.612 | 0.028 | 21.75 | <0.001 | 0.557 – 0.668 |
|  | Self-Criticism | 0.541 | 0.087 | 6.20 | <0.001 | 0.370 – 0.712 |
|  | Social Withdrawal | 0.671 | 0.026 | 26.09 | <0.001 | 0.620 – 0.721 |
| **Internalizing Behaviour** | Emotional Problems | 0.485 | 0.048 | 10.21 | <0.001 | 0.392 – 0.578 |
|  | Peer Problems | 0.435 | 0.043 | 10.22 | <0.001 | 0.352 – 0.519 |
| **Externalizing Behaviour** | Conduct Problems | 0.758 | 0.055 | 13.81 | <0.001 | 0.651 – 0.866 |
|  | Hyperactivity | 0.433 | 0.045 | 9.73 | <0.001 | 0.346 – 0.521 |
| **Anxiety (RCMAS-2)** | Physiological Anxiety | 0.690 | 0.024 | 28.31 | <0.001 | 0.642 – 0.738 |
|  | Worry/Oversensitivity | 0.813 | 0.021 | 38.81 | <0.001 | 0.772 – 0.854 |
|  | Social Concerns/Concentration | 0.802 | 0.028 | 28.86 | <0.001 | 0.748 – 0.857 |

**Supplementary Table 3: Reliability and Measurement Model Adequacy**

| **Latent Construct** | **Cronbach’s α** |
| --- | --- |
| Adaptive Coping | 0.78 |
| Maladaptive Coping | 0.81 |
| Internalizing Behaviour | 0.69 |
| Externalizing Behaviour | 0.73 |
| Anxiety (RCMAS-2 domains) | 0.86 |
